# Equine enteroid-derived monolayers recapitulate key features of parasitic intestinal nematode infection

**DOI:** 10.1186/s13567-024-01266-1

**Published:** 2024-02-27

**Authors:** Stina Hellman, Frida Martin, Eva Tydén, Mikael E. Sellin, Albin Norman, Bernt Hjertner, Pia Svedberg, Caroline Fossum

**Affiliations:** 1 Department of Biomedical Sciences and Veterinary Public Health, Swedish University of Agricultural Sciences, SLU, P.O. Box 7028, 750 07 Uppsala, Sweden; 2 Science for Life Laboratory, Department of Medical Biochemistry and Microbiology, Uppsala University, Uppsala, Sweden; 3 Vidilab AB, P.O. Box 33, 745 21 Enköping, Sweden

**Keywords:** Equine, organoid, enteroid, enteroid-derived, monolayer, nematode, larvae, intestine, in vitro

## Abstract

Stem cell-derived organoid cultures have emerged as attractive experimental models for infection biology research regarding various types of gastro-intestinal pathogens and host species. However, the large size of infectious nematode larvae and the closed structure of 3-dimensional organoids often hinder studies of the natural route of infection. To enable easy administration to the apical surface of the epithelium, organoids from the equine small intestine, i.e. enteroids, were used in the present study to establish epithelial monolayer cultures. These monolayers were functionally tested by stimulation with IL-4 and IL-13, and/or exposure to infectious stage larvae of the equine nematodes *Parascaris univalens*, cyathostominae and/or *Strongylus vulgaris.* Effects were recorded using transcriptional analysis combined with histochemistry, immunofluorescence-, live-cell- and scanning electron microscopy. These analyses revealed heterogeneous monolayers containing both immature and differentiated cells including tuft cells and mucus-producing goblet cells. Stimulation with IL-4/IL-13 increased tuft- and goblet cell differentiation as demonstrated by the expression of DCLK1 and MUC2. In these cytokine-primed monolayers, the expression of MUC2 was further promoted by co-culture with *P. univalens*. Moreover, live-cell imaging revealed morphological alterations of the epithelial cells following exposure to larvae even in the absence of cytokine stimulation. Thus, the present work describes the design, characterization and usability of an experimental model representing the equine nematode-infected small intestinal epithelium. The presence of tuft cells and goblet cells whose mucus production is affected by Th2 cytokines and/or the presence of larvae opens up for mechanistic studies of the physical interactions between nematodes and the equine intestinal mucosa.

**Supplementary Information:**

The online version contains supplementary material available at 10.1186/s13567-024-01266-1.

## Introduction

Stem cell-derived intestinal organoid cultures provide unique opportunities for detailed studies of the host–pathogen interplay at the intestinal epithelium of various animal species [1–5]. The small 3-D structures of polarized intestinal cells at various stages of differentiation, forming a budding lumen with closed ends, allow studies of enteric infections with a minimal use of experimental animals. To date, most published work on organoid-pathogen interactions involves viruses, bacteria and protozoa [6–8], while the organoid technology has only just begun to be explored for studies of gastrointestinal (GI) nematodes [9–12]. Gastrointestinal nematode infections cause health problems in both human and veterinary medicine that tend to aggravate with the development of anthelminthic resistance as reported for decades [13, 14]. Despite that, the development of alternative treatments and prophylactic measures are slow, partly due to lack of feasible in vitro models to study interactions between GI nematodes and their hosts. Many equine parasites enter their host via ingestion followed by colonisation or penetration of the intestinal epithelium. Thus, examinations of physical interactions between the parasite and the host´s epithelial defence mechanisms are needed to better understand and prevent common equine nematode infections, such as those with *Strongylus vulgaris*, cyathostomins and *Parascaris univalens*. For that purpose, the establishment of equine 3-D intestinal organoids, enteroids, was progressed to enteroid-derived 2-D monolayers allowing apical interaction with GI nematodes under the influence of basolateral stimuli.

The intestinal defence mechanisms involve mucus production by Goblet cells, defensin production by Paneth cells, hormone secretion by neuroendocrine cells and cytokine production by enterocytes and tuft cells dispersed in the epithelium. When sensing parasite antigens these cells will alarm immune cells and regulate the ensuing expulsion response [15]. Initiation of this response is mainly orchestrated by enterocytes and tuft cells that react to nematodes and/or their products by releasing the alarmins IL-25, IL-33 and thymic stromal lymphopoietin (TSLP). These cytokines are commonly produced in response to mucosal insults, which in turn activate T helper type 2 (Th2) immune cell polarization with production of IL-4 and IL-13. Together, these cytokines activate a series of effector functions collectively termed the “weep and sweep” response. The main characteristics of this response are expansion of tuft- and goblet cell populations, increased mucus production and increased intestinal peristalsis to trap and expel worms [15, 16]. Thus, to reflect these early events of nematode intestinal infection in vitro, a relevant organoid model system is dependent on the presence and flexible differentiation of appropriate cell lineages, such as tuft- and goblet cells.

Both the large size of GI nematode larvae and the natural inwards-facing polarity of 3-dimensional (3D) organoids pose challenges in using traditional basal-out organoids to model host-parasite interactions at the natural site of infection. Additionally, nematodes of both sheep, *Teladorsagia circumcincta*, and cattle, *Ostertagia ostertagi*, were shown to burrow into the 3D organoid lumen from the basolateral side although this is not occurring in vivo [11, 12]. Moreover, the protozoan parasite *Trypanosoma cruzi* could invade murine colon organoids (colonoids) from both the apical and basolateral surface [17]. These organoid models also illustrated interesting physical in vivo effects. Though organoid entry may deviate from in vivo infection pathways, organoids do recapitulate cellular responses such as swelling when exposed to *O. ostertagi* and intracellular replication by *T. cruzi*. However, this may be difficult to reproduce for parasite species that exclusively infect apically, especially when focusing on epithelial invasion and how it can be hindered.

To expose the apical surface of the epithelium, 3D organoids can be used to generate 2D monolayers [18, 19], which facilitates delivery of infectious agents and/or their products to the apical compartment. This approach was recently used for infecting monolayers of murine ceacal organoids with *Trichuris muris* larvae, providing novel insights into the early stages of intestinal whipworm invasion [10]. Furthermore, growing organoid monolayers in transwell culture systems allows for separate apical and basolateral manipulation of the epithelium making it possible to mimic the environmental conditions encountered by epithelial cells in vivo [reviewed in 19]. It should however be noted that the different conformations in which intestinal organoids can be grown vary greatly in dimensions and cell differentiation patterns. For example, growing organoids as monolayers tend to promote an immature state rather than full differentiation, which can potentially affect the ability of pathogens to interact with the epithelial cells [6]. Thus, to truly recapitulate parasite epithelial cell interactions, each type of organoid formulation must be characterized regarding the presence and functional activity of defined cell populations.

The present study was undertaken to explore the utility of previously established equine small intestinal 3D enteroids and enteroid-derived 2D monolayers [3], focusing on key epithelial functions in the response to nematode infection. To model the relevant infection conditions, the equine enteroid monolayers were basolaterally stimulated with the Th2 polarizing cytokines IL-4 and IL-13 and/or exposed to the infectious larval stage (L3) of three equine GI nematodes, namely cyathostomins, *P. univalens* and *S. vulgaris.* Effects, including the presence of tuft cells and mucus-producing goblet cells, were studied using transcriptional analysis combined with histochemistry, immunofluorescence imaging and scanning electron microscopy (SEM). Finally, a recently developed method for live-cell imaging of enteroid monolayers [20] was adapted for the present experimental set-up, enabling differential interference contrast (DIC) microscopy of the apical monolayer surface during exposure to nematode larvae.

## Materials and methods

### Culture of equine enteroids

Enteroids were cultured from cryopreserved enteroids generated in a previous study from sections of equine mid-jejunum [3]. The frozen enteroid material originated from two individual horses (Swedish warmblood mares, 10 and 14 years old) and the cultures were set up using a previously published protocol [3]. Briefly, frozen enteroids were thawed, washed, fragmented and suspended in ice-cold Matrigel (Corning™) supplemented with the recombinant human growth factors Noggin (100 ng/mL; Peprotech), R-spondin (500 ng/mL; Peprotech), EGF (50 ng/mL; Corning™), Wnt3a (100 ng/mL; Peprotech), Y-27632 (10 µM; Peprotech), SB202190 (10 µM; Tocris Bioscience™), LY2157299 (500 nM; A ChemBlock) and CHIR99021 (2.5 µM; Tocris Bioscience™). Approximately 20–40 crypts in 50 µL Matrigel were plated as domes in 24-well plates (Nunc) and covered by 0.5 mL enteroid growth medium (EGM), i.e. DMEM/F12 containing 1 × GlutaMAX (Gibco™), 200 IU/mL penicillin, 100 µg/mL streptomycin and 10 mM HEPES (Invitrogen, CA, USA), 1 × N-2 and 1 × B-27 (Gibco™). Growth factors as specified above were added every second day and the enteroids were propagated by passage every 4–5 days.

### Establishment of equine enteroid monolayers

Equine enteroid monolayers were generated as previously described [3]. Briefly, enteroids at day 4–5 after passage were disrupted to a single cell suspension by 10 min incubation in 1 × TrypLE Express Enzyme (Gibco™) at 37 °C and mechanically dissociated by pipetting. The TrypLE dissociation was stopped by addition of four volumes ice-cold EGM containing 5% fetal calf serum (FCS; Invitrogen) and 10 µM Y-27632 and the fragmented enteroids thereafter resuspended in EGM supplemented with the growth factors as specified above.

**Figure 1:**
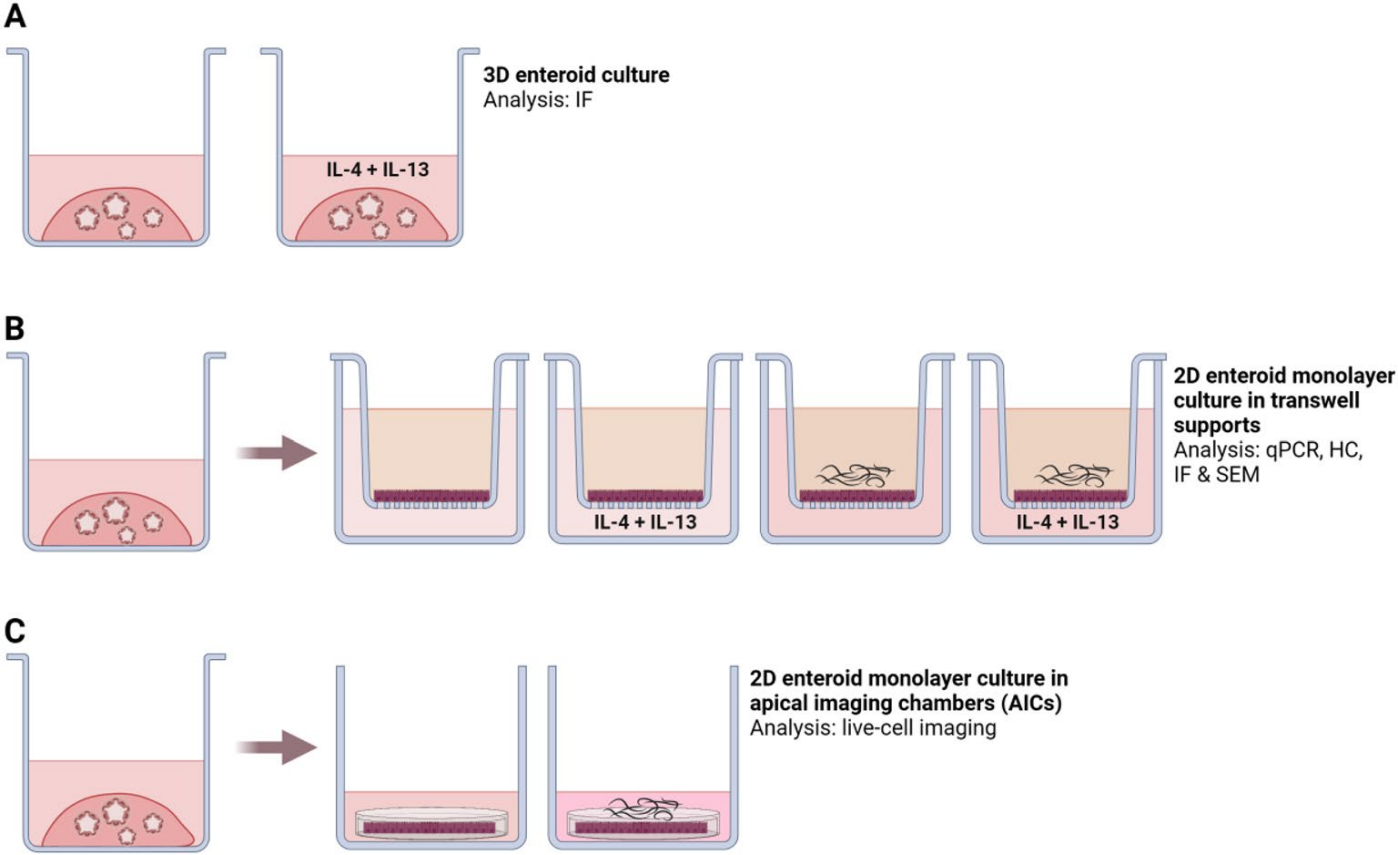
**Illustration of the experimental setup**. Equine 3D enteroids and 2D monolayers were generated from tissues originating from two individual horses. **A** Equine 3D enteroids from horse 1 cultured in plain growth medium or stimulated with eqIL-4 and IL-13 were used to optimize the labelling conditions for immunofluorescence microscopy (IF). **B** Equine 3D enteroids from horse 1 and 2 were disrupted to single cells, cultured as 2D monolayers on transwell supports and monitored by TEER. The monolayers were grown in plain growth medium or in the presence of eqIL-4/IL-13 before exposure to different combinations of *P. univalens*, cyathostomin or *S. vulgaris* larvae. Gene expression of cytokines/chemokines and cell lineage markers were examined by qPCR analysis, and verified by IF and SEM. **C** To enable live-cell imaging during exposure to nematode larvae, enteroid monolayers originating from horse 1 and 2 were cultured in AICs built to optimize the optical conditions for DIC microscopy of the apical epithelial surface. These AIC-grown monolayers were exposed to *P. univalens*, cyathostomin and *S*. *vulgaris* larvae and compared to parallel controls. Illustration created with https://www.BioRender.com.

Monolayers were cultured on semi-permeable transwell polycarbonate inserts with a 0.4 µm pore size for 12-well plates (Thermo Fisher; Figure 1). The transwell membranes were prepared by 1–2 h incubation in a 1:30 mix of Matrigel and EGM and then air-dried for 10 min prior to use. The enteroid cells were seeded at a concentration of 40–50 000 cells/cm^2^ and cultured in 0.5 mL growth factor-supplemented EGM in the upper chamber and 1.5 mL in the lower chamber of the well. Half of the medium volume (0.25 and 0.75 mL, respectively) was replaced every second day. The monolayer integrity was monitored every 2–3 day by measuring the trans-epithelial electrical resistance (TEER) using an Epithelial Volt-Ohm Meter (Millicell ERS-2, Millipore). Monolayers reaching a TEER of > 800 Ω*cm^2^ were characterized by histological stainings and used for larval exposure experiments. In the present experimental set-up, this condition was achieved after 5–6 days of culture. The enteroids and/or enteroid monolayers were exposed to various stimuli and characterized as outlined in Figure 1 and specified below.

### Preparation of cyathostomin, *S. vulgaris* and *P. univalens* third stage larvae

Eggs of cyathostomin, *S. vulgaris* and *P. univalens* larvae (L3) were prepared from fecal samples collected from naturally infected privately owned horses, using previous published protocols and identification cues [21, 22]. Before use, L3s of all three species were decontaminated by 24 h incubation in PBS containing 400 IU/mL penicillin, 200 IU/mL streptomycin, 1 µg/mL amphotericin and 30 µg/mL polymyxin B (Sigma-Aldrich, USA). *Strongylus vulgaris* and cyathostomin L3s were thereafter exsheated by 4–5 min incubation in 0.2% sodium hypochlorite pre-warmed to 37 °C, then repeatedly washed in PBS at RT. All centrifugation steps were performed for 5 min at 50×*g*. For all preparations, the endotoxin content was below 0.23 IU/mL as determined by the LAL assay (Pierce™).

### Combined apical and basolateral stimulation with Th2-polarizing cytokines and nematode larvae

Transwell-grown equine enteroid monolayers were primed basolaterally with a combination of 25 ng/mL equine yeast-derived recombinant IL-4 and 25 ng/mL IL-13 (eqIL-4/IL-13; Kingfisher Biotech, Inc) at the second day of culture and kept in parallel with unstimulated monolayers. After a total culture time of 5–6 days, both types of cultures were apically subjected to ~20 living L3 of *P. univalens*, *S. vulgaris* or cyathostomins, or maintained as untreated controls. After 20 or 48 h incubation, the monolayers were harvested and compared by qPCR analysis or immunofluorescence imaging, respectively (Figure 1B).

### RNA isolation and cDNA synthesis

RNA was extracted by combining Trizol (Invitrogen, USA) with the E.Z.N.A total RNA kit (Omega Biotek, USA), as previously described [22]. To ensure enough RNA for the cDNA synthesis, monolayers intended for qPCR were set up in duplicates that were pooled at harvest. To make cDNA, 1.2 µg of RNA was treated with RQ1 RNAse-free DNAse (Promega) followed by cDNA synthesis using the GoScript Reverse Transcription System (Promega). To ensure that all genomic DNA had been eliminated, -RT controls were run in parallel. The samples were diluted 1:5 in nuclease-free H_2_O and stored at −20 °C until use.

### qPCR analysis

The expression of cell lineage markers EPCAM (epithelial cells), PCNA (proliferative cells), SOX9 (proliferative- and stem cells), LYZ (Paneth cells), CGA (enteroendocrine cells), MUC2 (goblet cells) and DCLK1 (tuft cells) was examined by qPCR using previously published primers [3]. Specific cytokine and chemokine transcript responses was quantified using primers for equine IL-5, IL-8, IL-18 and TGF-β [3, 22, 23]. In addition, sequences for CXCL10 (ENSECAT00000013951) and MIF (ENSECAG00000012792) were identified in the equine genome (GCA_002863925.1) in ENSEMBL [24]. Primers were designed to flank intronic sequences using Primer3web [25] and ordered from Eurofins Genomics (Galten, Denmark). Primers were optimized for qPCR regarding annealing temperature and concentration to a 95–100% efficiency (Additional file 1). In addition, PCR products were confirmed by gel electrophoresis and by Sanger sequencing. Duplicate reactions of 2 µL cDNA in 23 µL qPCR mix (i.e. Qiagen Quantitect SYBR Green PCR mix + nuclease free H_2_O + primers) were run on a CFX96 Touch PCR machine (Bio-Rad), starting at 95 °C for 15 s followed by 40 cycles of 95 °C for 15 s, the assay specific annealing temperature for 30 s and 72 °C for 30 s. Based on a previous evaluation of equine enteroid samples [3], the three reference genes GAPDH, HPRT and SDHA were selected for normalization of data. The fold change value for the gene of interest was calculated by normalization to the geometric mean for the reference genes followed by calibration to the untreated control [26]. Differences in gene expression between treatments were calculated on ΔΔCt values using the one-way ANOVA followed by Dunnett’s multiple comparisons test or the paired Student’s *T*-test using the Graph Pad software (Prism 7.0). *P*-values < 0.05 were regarded as significant. When indicated, gene expression data is reported as mean ± SD.

### Histological sectioning and staining of enteroid monolayers

Transwell-grown equine enteroid monolayers cultured in EGM for 5–6 days were fixed with Methanol-Carnoy’s solution (60% methanol, 30% chloroform and 10% glacial acetic acid) to ensure preservation of mucus. The membranes were cut from the plastic frame and divided into three strips, approximately 4 mm wide. The membrane strips were placed between foam pads in embedding cassettes, dehydrated overnight (routine program, 13–14 h) in a tissue processor (ThermoFisher Excelsior) and transferred to an embedding station. To prepare monolayer cross sections, the strips were oriented perpendicularly and embedded in paraffin in an embedding base mold. After cooling, the samples were sectioned in a rotary microtome (Microm/ThermoFisher) into 4 µm sections and placed on slides (SuperFrost Plus). To visualize acidic and neutral mucins, the slides were stained with the Alcian Blue (AB) and Periodic acid-Schiff’s (PAS) technique, respectively, using slightly modified standard protocols. Briefly, the slides were dried overnight at 37 °C followed by incubation at 60 °C, deparaffinized, rehydrated and stained in AB, pH 2.5. The slides were then oxidized in 0.5% periodic acid and stained in Schiff’s reagent using Mayer’s haematoxylin as a nuclear counterstain. Finally, the samples were dehydrated, cleared and mounted with coverslips.

### Immunofluorescence staining of enteroids and enteroid monolayers

To visualize tuft cells and mucus-producing goblet cells, rabbit anti-human DCLK1 (ab31704; Abcam), diluted 1:1000, previously referred to as DCAMKL1 [27] or rabbit anti-MUC2 (PA5-79,702; Thermo Fisher), diluted 1:100, were used as primary antibodies. The same secondary antibody, goat anti-rabbit IgG labelled with Alexa Fluor™ 488 (1:600; ab150077; Abcam) was used for both MUC2 and DCLK1 staining. The labelling conditions for each antibody were set on enteroids harvested at day 4 after passage, either cultured in plain EGM or in EGM supplemented with eqIL-4/IL-13 (25 ng/mL) for the last 48h of culture. The enteroids were harvested in cell recovery solution, washed three times in PBS + 0.1% BSA and fixed in 4% paraformaldehyde for 30 min at RT. The fixed enteroids were permeabilized and blocked with staining buffer, i.e. 1 × BD Cytoperm™ permeabilization buffer plus (BD Biosciences) in PBS + BSA, for 30 min at RT. The enteroids were incubated with primary and secondary antibodies diluted in staining buffer for 24 h each at RT. In between incubations, the enteroids were washed three times in staining buffer. Finally, the stained enteroids were resuspended in a small volume (< 1 mL) of PBS and placed on a microscope slide immediately before imaging.

Transwell-grown equine enteroid monolayers were fixed in Methanol-Carnoy’s solution for 30 min at RT followed by a gentle rinse in PBS + BSA. The monolayers were incubated with anti-MUC2 diluted in PBS + BSA for 4 h at RT, gently washed and thereafter incubated with the secondary antibody for another 4 h. Monolayers were also stained with Alexa Fluor^®^ 488 Phallodin (Sigma Aldrich) for detection of actin filaments and counterstained with DAPI (Bio-Rad Laboratories, Inc) according to the manufacturer’s protocols. Stained membranes were cut out from the inserts and mounted on glass microscope slides using ProLong Diamond Antifade Mountant (Invitrogen).

### Confocal laser scanning microscopy

Fluorescence images and z-stacks were captured using an inverted LSM800 laser scanning confocal microscope equipped with 405, 488, 560 and 633 nm lasers, 10x/0.3 NA, 40x/1.4 NA or 63x/1.2 NA water emulsion objectives and the Zen black acquisition software (Carl Zeiss). Z-stacks were acquired in 25 z-sections at 0.66 µm intervals and displayed as maximum intensity projections. Images of enteroids stained for DCLK1 were acquired using 488 excitation (green channel) but displayed in red color. DCLK1 cell counts were determined from images in a single z-plane. Brightness and contrast were adjusted in the Zen blue (Zeiss) or Fiji [28] software and all images belonging to the same group were acquired and processed using the same settings.

### Scanning electron microscopy

SEM analysis was performed on transwell-grown equine enteroid monolayers cultured for 8 days and after 48h exposure to a cocktail of 15–20 each of *P. univalens*, *S. vulgaris* and cyathostomin L3s and compared to untreated control cultures. The monolayers were washed in PBS and fixed by 24 h incubation at 4 °C in 2.5% glutaraldehyde (Sigma) in 0.1 M PHEM buffer (60 mM piperazine-N, N9-bis(2-ethanesulfonic acid), 25 mM HEPES, 10 mM EGTA and 4 mM MgSO4) at pH 6.9. The samples were prepared for SEM by repeated dehydration in graded ethanol and critical point drying (Leica EM CPD300) and then coated with 5-nm platinum (Quorum Q150T-ES sputter coater). Images were captured by a field emission scanning electron microscope (Carl Zeiss Merlin) using in-lens and in-chamber secondary electron detectors at accelerating voltage of 4 kV and probe current of 100 pA.

### Live-cell imaging of equine enteroid monolayers upon exposure to nematode larvae

A method to improve the optical conditions for live-cell imaging, described in detail for human enteroid monolayers [20] was applied. In brief, equine enteroid monolayers were grown on alumina Whatman Anodisc membranes (13-mm-diameter with 0.2 µm pores) placed within custom-designed 3D-printed holders denoted “Apical Imaging Chambers” (AICs). To prepare the surface for Matrigel coating, the alumina membranes were pre-treated by 1 h soaking in 20% H_2_O_2_ at RT followed by a rinse in sterile distilled H_2_O (dH_2_O) and 5 min incubation in 0.1 mg/mL poly-L-lysine (Sigma-Aldrich). The poly-L-lysine coated membranes were air-dried o.n., soaked in Matrigel diluted 1:30 in dH_2_O for 1 h and thereafter air-dried o.n. again. After coating, the membranes were mounted within the AICs and placed in 12-well plates (Nunc, Thermo Fisher Sci). Approximately 25–35 000 enteroid cells in 150 µL growth factor supplemented EGM were seeded into the top compartment of the AICs and 600 µL growth factor supplemented EGM was added to the bottom of the well. After the cells had adhered to the membrane, an additional 250 µL medium was added to cover the AIC. Thereafter, half the medium volume in the well (500 µL) was changed every 2–3 days. The AIC monolayers were kept in culture for 5–6 days before exposure to nematode larvae and microscopy. Live-cell imaging was performed using a custom-built upright microscope described in detail in van Rijn et al. [20]. Briefly, the microscope was equipped with a heated 60 × /1.0 NA objective (Nikon CFI APO NIR, 2.8 mm WD), a differential interference contrast (DIC) oil condenser (Nikon d-CUO, 1.4 NA), and placed in a microscope chamber maintaining 37 °C, 5% CO_2_. The AIC-monolayers were placed in 35-mm glass-bottom dishes (Cellvis, Mountain View, CA, USA) containing 3 mL growth factor supplemented EGM and exposed to a cocktail of 15–20 each of *P. univalens*, exsheated *S. vulgaris* and exsheated cyathostomin L3s. Nematode-exposed and non-exposed control cultures were monitored for 3 days and DIC images were acquired at 0, 24, 48 and 72 h. To obtain an even field of illumination, the images were processed by subtracting a Gaussian blurred projection (30-pixel sigma) from the original images using the Fiji software [28].

## Results

### Characterization of equine enteroid monolayers

**Figure 2:**
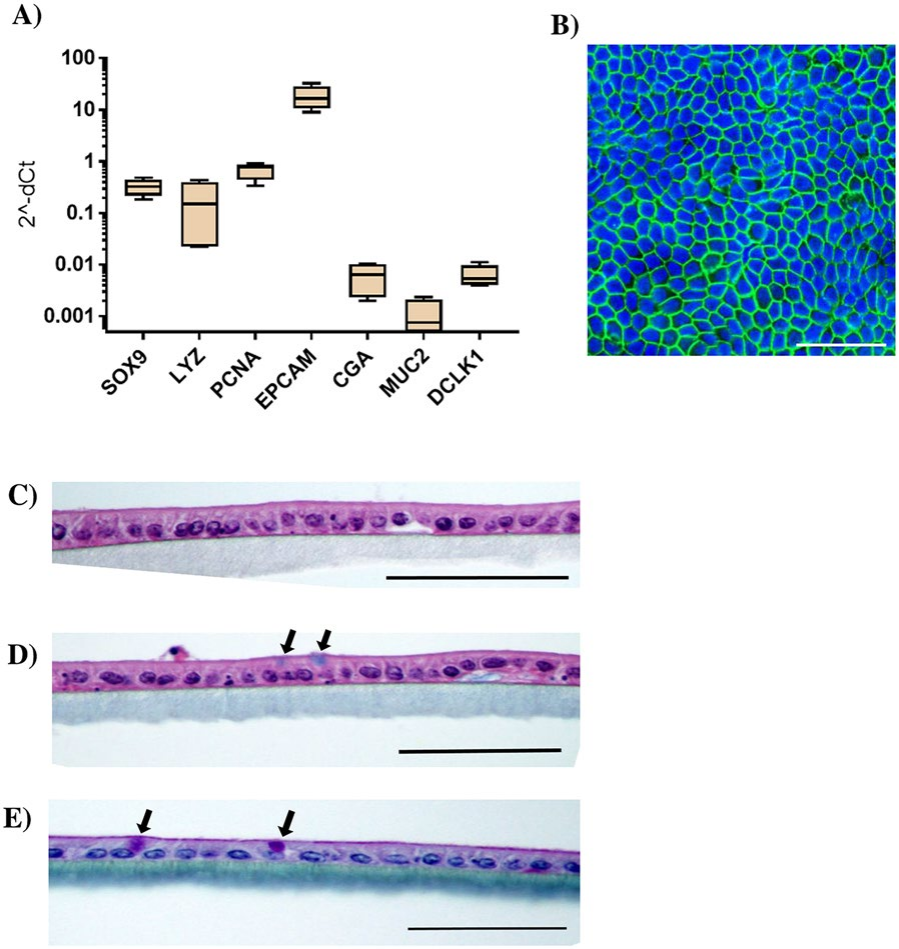
**Characterisation of equine enteroid monolayers**. **A** Expression of cell lineage markers SOX9, LYZ, PCNA, EPCAM, CGA, MUC2 and DCLK1 in enteroid monolayers after 5–6 days culture on transwell supports. The gene expression was normalized to the geometric mean for the reference genes HPRT, SDHA and GAPDH and is presented as 2^−ΔCt^ The results are generated from monolayers originating from two horses, each used in two separate experiments, giving a sample size of *N* = 4. **B** Representative confocal image of transwell-grown enteroid monolayers stained for nuclei (DAPI) and F-actin (phalloidin). **C** Monolayer cross-sections stained with HE to visualize monolayer structure. **D** Monolayer cross-sections stained with AB to detect the presence of acidic mucins and **E** with AB-PAS to detect neutral mucins. Mucus-containing cells are indicated by arrows. Scale bars = 50 µm.

Single cells obtained after disruption of the equine enteroids established confluent monolayers after 2–3 days when cultured on semipermeable transwell membranes (Figure 1). After a total culture time of 5–6 days, TEER values of at least 800 Ω*cm^2^ were recorded. At this time, the equine enteroid monolayers expressed the cell-lineage marker genes SOX9, LYZ, PCNA, EPCAM, CGA, MUC2 and DCLK1 indicating the presence of stem cells, immature proliferative cells, Paneth cells, absorptive epithelial cells, enteroendocrine cells, goblet cells and tuft cells, respectively (Figure 2A). Actin staining of the monolayers (Figure 2B) and HE staining of cross-sections (Figure 2C) demonstrated two-dimensional growth with no gaps or cellular overlap. Staining with AB and AB-PAS indicated cells containing acidic (Figure 2D) and neutral (Figure 2E) mucins as well as a 0.5–0.8 µm thick mucin lining at the apical brush border (Figure 2E). Thus, the gene expression data indicated the establishment of a heterogenous enteroid monolayer containing also secretory cell types, which was further supported by mucin staining.

### Th2 cytokines promote tuft- and goblet cell differentiation in equine enteroids and enteroid monolayers

**Figure 3:**
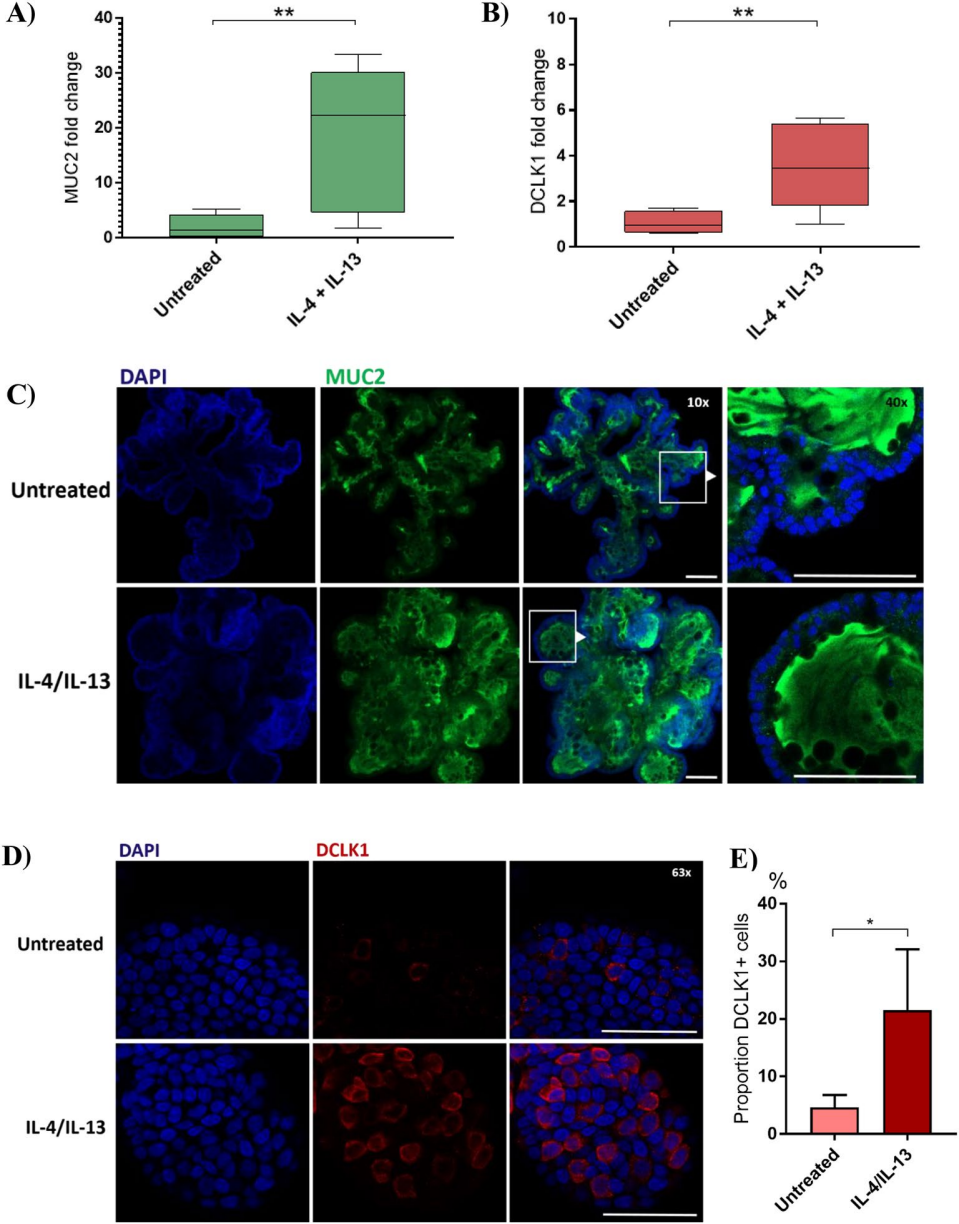
**Expression of MUC2 and DCLK1 in enteroid 2D monolayers and 3D enteroids stimulated with eqIL-4/IL-13.** Relative gene expression of **A** MUC2 and **B** DCLK1 transcripts in transwell-grown equine enteroid monolayers basolaterally stimulated with eqIL-4/IL-13 and compared to untreated controls. The gene expression was normalized to the geometric mean for the reference genes HPRT, SDHA and GAPDH and compared to the mean ΔΔCt of the untreated controls. The monolayers were generated from two individual horses and used in two (horse 1) and three (horse 2) separate experiments, giving a sample size of *N* = 5. **C, D** Confocal images of equine 3D enteroids cultured in plain growth medium or stimulated with eqIL-4/IL-13 for 48 h before staining with DAPI (blue) and **C** MUC2 (green) or **D** DCLK1 (red). Representative images from one horse. **E** Proportion of cells positive for DCLK1 staining. Mean ± SD of 4 technical replicates. Scale bars = 100 µm for C and 50 µm for D. *P < 0.05, **P < 0.01.

Transwell-grown equine enteroid monolayers were next basolaterally stimulated with eqIL-4/IL-13 and evaluated for alterations in epithelial cell differentiation. This stimulation resulted in a significantly higher gene expression of the goblet cell marker MUC2 (*P* = 0.0015; Figure 3A) and the tuft cell marker DCLK1 (*p* = 0.004; Figure 3B) compared to the unstimulated controls. In contrast, the gene encoding LYZ was downregulated after stimulation (Table 1). The expression of CGA, EPCAM, SOX9 and PCNA was essentially unaffected by this cytokine stimulation (Table 1).

**Table 1:** **Alterations in gene expression after basolateral cytokine stimulation and/or apical exposure to nematode larvae**.

Exposed to	IL-4 + IL-13	*P. univalens*	*P. univalens* + IL-4 + IL-13	Cyathostomins	Cyathostomins + IL-4 + IL-13	*S. vulgaris*	*S. vulgaris* + IL-4 + IL-13
Gene name							
SOX9	0.54 ± 0.08	1.06 ± 0.08	0.51 ± 0.09	1.05 ± 0.16	0.57 ± 0.07	0.98 ± 0.10	0.52 ± 0.22
LYZ	0.23 ± 0.23	0.74 ± 0.18	0.13 ± 0.05	0.93 ± 0.18	0.17 ± 0.06	0.86 ± 0.24	0.24 ± 0.12
PCNA	0.61 ± 0.14	0.96 ± 0.12	0.44 ± 0.08	0.94 ± 0.03	0.54 ± 0.10	1.10 ± 0.09	0.69 ± 0.18
CGA	0.54 ± 0.11	1.07 ± 0.15	0.67 ± 0.13	1.06 ± 0.32	0.58 ± 0.16	1.18 ± 0.31	0.52 ± 0.12
EPCAM	0.89 ± 0.17	1.13 ± 0.04	0.96 ± 0.22	1.05 ± 0.12	0.86 ± 0.16	1.02 ± 0.09	0.72 ± 0.06
IL-5	1.02 ± 0.49	1.01 ± 0.31	1.15 ± 0.27	1.04 ± 0.20	1.21 ± 0.42	0.71 ± 0.32	0.59 ± 0.28
IL-8	2.11 ± 1.24	1.11 ± 0.35	1.82 ± 1.12	1.02 ± 0.16	2.82 ± 2.50	0.90 ± 0.19	1.92 ± 1.32
TGF-β	0.55 ± 0.13	1.24 ± 0.12	0.78 ± 0.27	1.10 ± 0.16	0.62 ± 0.26	0.84 ± 0.32	0.49 ± 0.17
IL-18	0.61 ± 0.20	1.19 ± 0.14	0.82 ± 0.14	1.02 ± 0.10	0.72 ± 0.12	0.78 ± 0.28	0.54 ± 0.25
CXCL10	1.21 ± 1.04	1.27 ± 0.27	1.52 ± 1.41	0.81 ± 0.43	1.39 ± 1.26	1.05 ± 0.69	0.85 ± 0.43
MIF	0.76 ± 0.18	1.14 ± 0.27	1.05 ± 0.15	1.21 ± 0.45	0.88 ± 0.22	0.78 ± 0.33	0.65 ± 0.21

Transwell-grown equine enteroid monolayers were primed basolaterally with IL-4 and IL-13, apically subjected to ~20 living L3 of *P. univalens*, *S. vulgaris* or cyathostomins, combination of basolaterally IL-4 and IL-13 and apically to L3 or kept as untreated controls.

After incubation, the monolayers were harvested and compared by qPCR analysis. The monolayers were generated from two individual horses, each subjected to apical and/or basolateral treatment in two separate experiments, giving a sample size of *N* = 4.

Relative expression (fold change ± SD) of cell lineage markers, cytokine- and chemokine genes was normalized to the geometrical mean for the reference genes (GAPDH, HPRT and SDHA) and calibrated to that in the controls.

To confirm the cytokine-induced mRNA expression of MUC2 and DCLK1 on protein level, immunofluorescence labeling conditions were elaborated using 3D enteroids grown in EGM supplemented with eqIL-4/IL-13 for 48 h and compared to unstimulated enteroids. Confocal laser scanning microscopy (Figure 3C) showed MUC2-positive staining in the enteroid lumen of both untreated and IL-4/IL-13 stimulated enteroids, illustrating mucus production by goblet cells (Figure 3C). The presence of tuft cells was confirmed by DCLK1 staining, which appeared primarily localized to the cytoplasmic area of the cells (Figure 3D). Compared to unstimulated enteroids, eqIL-4/IL-13-stimulated enteroids featured a higher number of DCLK1-positive cells (Figure 3E).

### Equine enteroid monolayer differentiation in response to *P. univalens,* cyathostomin or *S. vulgaris* infection in the absence and presence of Th2 cytokine stimulation

To further model the conditions of nematode infection, transwell-grown equine enteroid monolayers were basolaterally primed with eqIL-4/IL-13 and thereafter apically exposed to the infective stage of *P. univalens*, cyathostomin, or *S*. *vulgaris* larvae. For comparison, parallel monolayer cultures were either apically exposed to larvae, basolaterally exposed to cytokines, or kept as untreated controls. After 20 h of larval exposure, expression of cell-lineage marker genes and a selected panel of cytokine and chemokine genes were analyzed by qPCR (schematic in Figure 1B).

**Figure 4:**
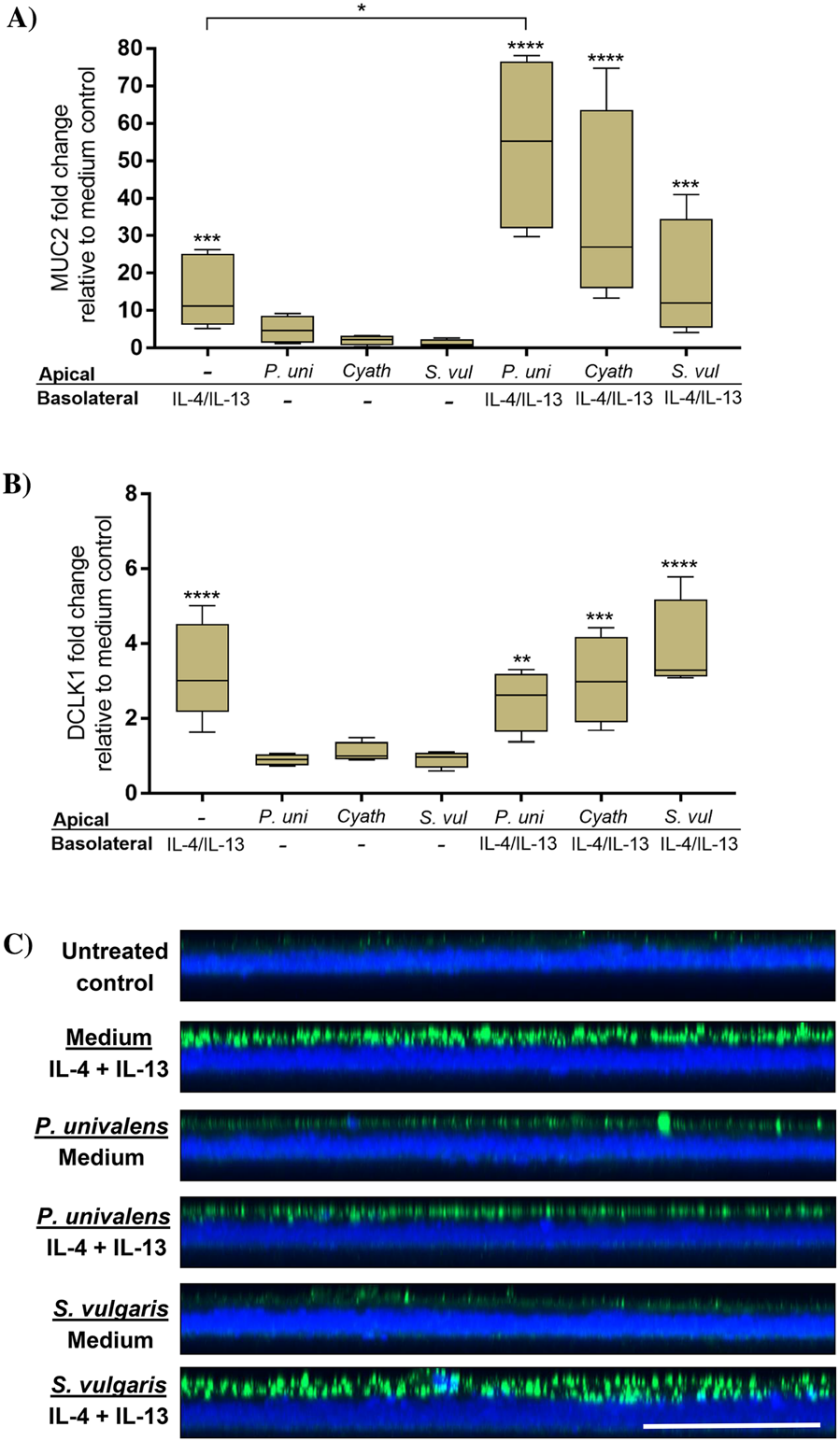
**Effect of eqIL-4/IL-13 and *P. univalens*, cyathostomin and *S. vulgaris* on the expression of MUC2 and DCLK1.** Relative gene expression of **A** MUC2 and **B** DCLK1 transcripts in monolayers basolaterally primed with eqIL-4/IL-13 before apically exposed to infective stage *P. univalens*, cyathostomin or *S. vulgaris* larvae for 20 h. As controls, parallel cultures were either apically exposed to larvae alone, basolaterally exposed to cytokines alone, or kept untreated. The gene expression was normalized to the geometrical mean for the reference genes (GAPDH, HPRT and SDHA) and calibrated to that in the untreated controls. The results were generated from monolayers originating from two individual horses, each subjected to apical and/or/basolateral treatments in two separate experiments, giving a sample size of *N* = 4. **C** Orthogonal slices of maximum intensity projections on the X–Z plane (25 z-stacks with 0.66 um apart) of enteroid monolayers stained for DAPI (blue) and MUC2 (green). Representative images from two individual experiments. Scale bars = 50 µm. *P < 0.05, **P < 0.01, ***P < 0.001 and ****P < 0.0001.

Exposure of unprimed monolayer cultures to *P. univalens*, cyathostomin or S. *vulgaris* did not induce differential expression of any of the cytokine, chemokine or cell-lineage marker genes tested (Figures 4A and B, Table 1). Interestingly, the expression of MUC2 was significantly higher in eqIL-4/IL-13-primed cultures exposed to *P. univalens* compared to cultures only stimulated with eqIL-4/IL-13 (*p* = 0.02; Figure 4A). A similar trend was also indicated in eqIL-4/IL-13-primed cultures subjected to cyathostomins*,* but this did not reach statistical significance (*p* = 0.08; Figure 4A). DCLK1 gene expression was upregulated at similar levels in all eqIL-4/IL-13-primed cultures, but seemed to remain unaffected by larval exposure (Figure 4B). Transcripts encoding IL-25, IL-33, TNF-α and TSLP were either below the detection limit of the qPCR used, or showed a low expression in only one of the technical replicates. Together, this data suggests that nematode exposure on its own has a marginal, or only a transient, effect on enteroid monolayer transcription as measured in bulk, but that larvae may boost IL-4/IL-13 induction of goblet cell MUC2 transcript levels.

The capability of the monolayers to respond with mucus production to nematode larvae and/or Th2 cytokines was further assessed by immunofluorescence staining and confocal microscopy. After 48 h exposure to *P. univalens* or S. *vulgaris* L3s, the enteroid monolayers were fixed in Carnoy’s solution and stained for MUC2. As illustrated in Figure 4C, Z-stack imaging indicated an elevated production of MUC2 in response to basolateral stimulation with eqIL-4/IL-13, whereas almost no MUC2-positive staining was detected in the untreated cultures (Figure 4C). Compared to the untreated control, a slight increase of MUC2-positive staining was noted in unprimed cultures exposed to *P. univalens* or *S. vulgaris*. The confocal images thus support the gene expression data (Figure 4A), further implying that the equine enteroid monolayers contain mucus-producing cells and that their frequency and secretion is affected by apical or basolateral stimulation(s) relevant to nematode infection.

### Scanning electron microscopy of equine enteroid monolayers reveals epithelial heterogeneity and nematode – epithelial cell interactions

**Figure 5:**
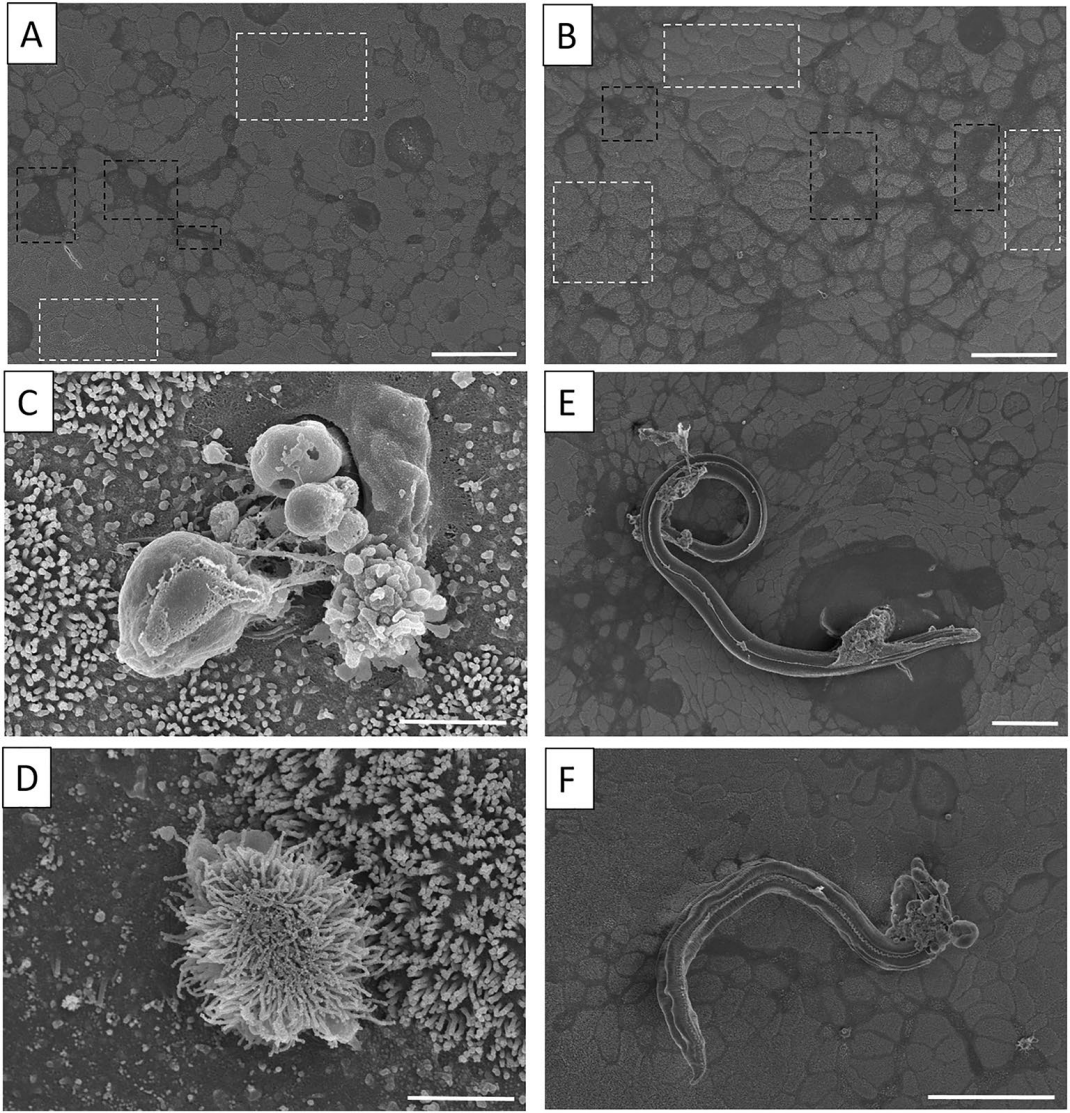
**Scanning electron microscopy of equine enteroid monolayers in the absence and presence of nematode larvae.** Representative SEM images of the apical surface of equine enteroid monolayers originating from one horse and kept as **A** untreated controls (*n* = 5) or **B** in co-culture with a mix of *P. univalens*, cyathostomin and *S. vulgaris* third stage larvae for 48 h (*n* = 3). The different degrees of microvilliated surfaces are indicated by dashed boxes showing areas with less (black) a more (white) dense microvilli. Both untreated and parasite exposed monolayers exhibited cellular structures indicative of (**C**) goblet cell orifices and (**D**) tuft cell microvilli. **E** Strongyle larvae and **F** *P. univalens* larvae atop the monolayer surface. Scale bars = 50 µm for **A**, **B**, **E**, **F** and 2 µm for **C**, **D**

Scanning EM revealed a heterogenous epithelial cell layer with different degrees of microvilliated surfaces in both larvae-exposed enteroid monolayers and untreated control cultures (Figures 5A and B). Structures resembling goblet cell orifices (Figure 5C) and apical microvillus tufts (Figure 5D) were found in both larval-exposed and untreated monolayers displaying similar morphologies. Thus, the formation of heterogenous equine enteroid monolayers encompassing also secretory cell lineages is supported by the SEM analysis. Despite repeated washing during preparation of the enteroid monolayers, L3 larvae were still found unevenly distributed across the monolayer surface in the infected samples (Figures 5E and F). Notably, these larvae frequently displayed accumulations of enteroid cells/cell debris attached at their anterior end.

### Live-cell imaging of equine enteroid monolayers during exposure to *P. univalens,* cyathostomin and *S. vulgaris* larvae highlights morphological alterations to the apical epithelial surface

**Figure 6:**
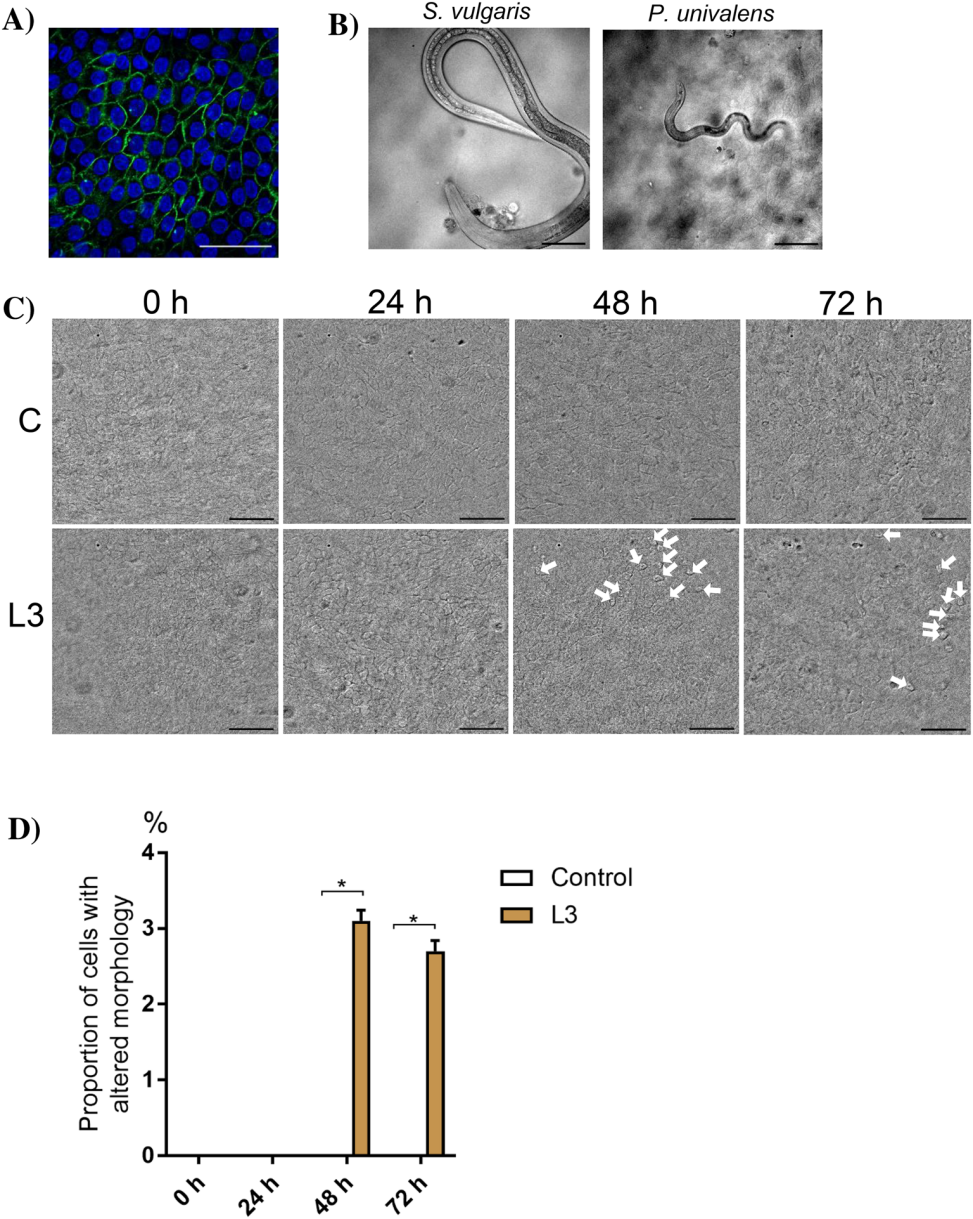
**Live-cell imaging of equine enteroid monolayers during exposure to *P. univalens,* cyathostomin and *S. vulgaris* L3. A** Confluent AIC-grown enteroid monolayers stained for nuclei (DAPI) and F-actin (phalloidin). **B** Snap-shot images showing nematode larvae atop the apical surface of equine enteroid monolayers. **C** Morphological patterns appearing as protruding cells with a ruffled surface (indicated by arrows) were observed after 48 h exposure to a mix of *P. univalens*, cyathostomin and *S. vulgaris* L3 (“L3”) but not in the control cultures (“C”). The figure shows a time-series of representative images from monolayers originating from one horse. **D** Proportion of cells displaying an altered apical surface morphology after 0, 24, 48 and 72 h incubation with or without larvae. Mean ± SD from two individual horses. Scale bars = 50 µm. *P < 0.05.

To visualize the nematode—epithelium interaction dynamics at the interface of infection, conditions for live-cell microscopy were elaborated for the equine enteroid monolayers in co-culture with infective stage nematode larvae (Figure 6). The cells successfully attached to the alumina membranes placed in AICs and generated a confluent polygonal cell layer within 2–3 days of culture (Figure 6A). After a total culture time of 5–6 days, the monolayers were subjected to co-infection of *P. univalens*, cyathostomin and *S. vulgaris* L3s*,* and monitored by DIC microscopy. High-resolution images of both larvae (Figure 6B) and the apical surface of the monolayers (Figure 6C) were recorded over a period of 0–72 h. During this time, larvae of all three species remained active and motile, appearing to probe the monolayer surface (Additional files 2 and 3). There were no signs of larval penetration or stable attachment to the epithelium. However, in agreement with the SEM analysis, pronounced epithelial cell/cell debris clustering was again noticed on the parasite’s anterior end (Figure 6B).

Of further interest, morphological changes appeared on the monolayers apical surface after 48 h of larval exposure and remained at 72 h post-challenge. These changes included the emergence of protruding cells with a “ruffled” surface structure clearly distinct from the typical enterocyte morphology, that were unevenly distributed over several patches of the monolayer. These patches were often found underlying one or several strongyle larvae and were completely absent in the control cultures (Figures 6C and D). Similar morphological patterns were seen in three separate experiments on monolayers originating from both horses (Figure 6D). This suggests that although we found no dramatic effects on enteroid monolayer transcription assessed in bulk at 20 h post-challenge (Figure 4), nematode exposure nevertheless impacts epithelial cell characteristics in affected regions.

## Discussion

Equine GI nematode infections are an increasing problem worldwide due to the rapid development of anthelmintic resistance in *P. univalens* and cyathostomins [29]. To find alternative treatments, a better understanding of the parasite interactions with the host intestinal barrier is needed. In that context, equine enteroids are attractive experimental models that can partially recapitulate the structure and function of the small intestinal epithelium [3, 4]. However, the large size of nematode larvae and the closed structure of traditional basal-out 3D enteroids complicate studies of the natural route of infection. To address this issue, equine enteroids were in the present study adapted into 2D monolayer cultures allowing easy administration of nematode larvae to the apical surface of the epithelium. These enteroid monolayers were functionally perturbed by basolateral stimulation with Th2 polarizing cytokines and/or apical exposure to the equine GI nematodes *P. univalens*, cyathostomins and *S. vulgaris*, and thereafter characterized for gene expression and morphology.

It was recently demonstrated that equine enteroids can be cultured in 3D conformation, as well as in open conformation as a 2D monolayer [3–5]. One consequence of plating out enteroid cells on a flat surface is that the organizational hierarchy with crypt-like domains rich in stem cells and villus-like regions containing differentiated cells is lost. Although there seems to be some degree of crypt-like spatial organization in enteroid monolayers of murine origin under some conditions [18, 30, 31], such cultures primarily contain immature cells with high proliferative activity that do not differentiate without modification of the culture medium [6, 32]. By removing or reducing the growth factors that stimulate the Wnt pathway, enteroids and enteroid monolayers of man and mice can be transformed from a stem-cell like to a more differentiated state [32]. In such cultures, dual Wnt and notch inhibition is generally needed for expansion of goblet cells [32, 33]. With this background, it was unexpected to find that the equine enteroid monolayers expressed the goblet cell marker MUC2 after 5–6 days of culture despite being kept in the presence of Wnt-stimulating factors. This is in consistence with our previous study that showed similar gene expression levels of MUC2 after 2–3 days of monolayer culture [3]. Further in support of this finding, presumed goblet cell orifices appearing as ring-shaped (crater)-like features containing secretory vesicles were in the present study observed by SEM. The combined expression of CGA, DCLK1, EPCAM, MUC2, SOX9 and PCNA further suggests that the established culture conditions upholds a population of proliferative cells with the capacity to differentiate into secretory cell lineages. A similar heterogenous gene expression profile was recently demonstrated for bovine enteroid monolayers using an in-house composed medium [34], emphasizing the need to optimize the culture conditions for each animal species and experimental setup.

To verify the presence of goblet cells, transwell-grown equine enteroid monolayers were carefully recovered, sectioned and stained for acidic and neutral mucins. This procedure verified a single cell layer interspersed with occasional mucin-containing cells. In addition, AB-PAS staining revealed a thin layer of mucins situated at the apical brush border, likely representing the membrane-bound mucins that build up the intestinal glycocalyx [35]. Similar staining procedures have illustrated changes in goblet cell distribution and mucin content in various equine intestinal disorders [36] and following the inflammatory response to equine cyathostomins [37]. Therefore, it seemed vital to assess if functional goblet cells are present and can be flexibly induced in equine enteroid monolayers aimed for GI-nematode research.

Even though intestinal mucus production is essential for the “weep and sweep” response occurring at expulsion of worms from the intestinal lumen [15, 27, 38, 39], the mucin components and/or associated proteins are likely also important for initial protection against invading larvae [15, 16]. Since the differentiation of goblet cells and their mucus production is promoted by IL-4 and IL-13, these type 2 cytokines were added into the growth medium of 3D enteroids or to the lower chambers of transwell-grown enteroid monolayers during the last 48 h of culture. Z-stack imaging of enteroid monolayers illustrated a marked increase in MUC2-positive staining after stimulation with eqIL-4/IL-13, compared to the weakly stained untreated control monolayers. In the 3D enteroids, intense staining of MUC2 was found in the lumen of both untreated and eqIL-4/IL-13-stimulated enteroids, likely reflecting the accumulation of mucus in these closed enteroid structures over time. Thus, mimicking the Th2 cytokine response typically evoked by GI nematode infection dramatically boosts mucin production by equine enteroid monolayers.

The production of IL-4 and IL-13 during nematode infection in vivo is mainly initiated by the alarmins IL-25, IL-33 and TSLP released by epithelial and stromal cells [40]. An important producer of IL-25 is the rather recently described chemosensory tuft cell (reviewed in [41]) that responds to GI nematodes and other intestinal insults [42]. In this context, mouse intestinal organoids have been indispensable in improving our understanding of the role of epithelial tuft cells in the initiation and regulation of type 2 immune responses against nematodes [9, 27, 39, 43, 44]. In accordance, the expression of DCLK1, marking tuft cells, was increased in both equine enteroids and enteroid monolayers by basolateral eqIL-4/IL-13 stimulation, as shown by immunofluorescence microscopy and gene expression analysis, respectively. Furthermore, cells with a tuft cell-resembling morphology, as described for other species [9, 45], were observed by SEM. Taken together, the gene expression data, immunohistochemical staining, confocal and scanning electron microscopy imply that the equine enteroid monolayers contain tuft cells and mucus-producing goblet cells whose frequency and expression is affected by basolateral stimulation with Th2 cytokines linked to nematode infection.

We have previously shown that equine enteroid monolayers respond to apical stimulation with viral and bacterial pathogen-associated molecular patterns (PAMPs) by inducing gene expression for anti- and pro-inflammatory cytokines [3]. However, no differential expression of these cytokines was observed in transwell cultures of equine enteroid monolayers after 20 h exposure to GI nematode larvae, regardless of whether the monolayers had been primed with eqIL-4/IL-13 or not. The only significant effect of nematode larvae was on the expression of MUC2 in monolayers that had been eqIL-4/IL-13-primed before exposure to *P. univalens* larvae. Effects on MUC2 production was also indicated by z-stack confocal imaging after 48 h exposure to *P. univalens* or *S. vulgaris* larvae. Larval effects in the absence of Th2 polarizing cytokines were further examined by SEM imaging. Although SEM revealed the presence of both goblet- and tuft cells, this type of imaging is not well suited for quantification of morphological alterations at various treatments of enteroid monolayers as it requires transfer and fixation of the monolayers on grids.

A current major limitation of the transwell culture system is its poor compatibility with live-cell imaging. To overcome this, a novel method for imaging pathogen interactions with human enteroid monolayers was recently demonstrated using *Salmonella enterica* Typhimurium and *Giardia intestinalis* as models for bacterial and protozoan infections, respectively [20]. This technology is built on custom imaging chambers that support monolayer growth while optimizing conditions for DIC microscopy to give sufficient optical contrast and resolution for tracing individual microbes atop the epithelium. To test if these AICs are compatible to study the infection dynamics of equine nematodes, conditions for co-culturing equine enteroid monolayers with cyathostomins, *P. univalens* and *S. vulgaris* L3s on AICs were established. During the entire co-incubation time of 72 h, the larvae remained motile across the monolayer surface. Despite this, no signs of stable larval attachment or invasion of the monolayer were observed. Notably, however, the larvae frequently accumulated epithelial cell debris at their anterior end while probing the monolayers. If this behaviour is relevant to nematode foraging, attempts at damaging the epithelial cell layer integrity, or some other aspect of the nematode infection cycle remains an intriguing question for future studies.

Moreover, the live-cell imaging revealed that epithelial cells with an altered apical morphology reproducibly appeared after 48 h of larval exposure, suggesting that either transient larval attachments or excretory/secretory (ES) products released at sites of contact affect the single cell characteristics of the epithelium. While the important role of nematode ES products in establishing and maintaining infections has been known for decades [46, 47], the secretome of equine nematodes and the effects of released ES products on the equine intestinal mucosa remain to be resolved. Although additional experiments are needed to evaluate the putative role of ES products in the present study, the results indicate that equine enteroid monolayers could serve as useful model for studying direct effects of ES products on the equine intestinal epithelium. Future studies should also explore if conditions can be optimized to visualize successful nematode traversal of the epithelial cell layer. This may include testing different states of cellular differentiation and/or increasing the pliability of the infection model, e.g. by culturing the enteroid monolayers atop collagen scaffolds [48, 49], or introducing an air–liquid interface [9, 50]. Regardless, the imaging technologies elaborated here will provide a meaningful basis for future studies of nematode infection dynamics at the intestinal epithelial barrier.

In conclusion, an experimental model representative of the nematode-infected equine small intestine that can be analyzed by various imaging techniques was established. These equine enteroid monolayers contain tuft cells and mucus-producing goblet cells whose differentiation and relative abundance can be controlled by addition of Th2 polarizing cytokines. Co-incubation with nematode larvae enables detailed studies of parasite-induced effects on the intestinal epithelium, demonstrating the potential for using enteroid monolayers as an in vitro tool to study host-nematode interactions in the equine gut.

### Supplementary Information

**Additional file 1. Table of novel primers for qPCR.**

**Additional file 2. Live-cell imaging of a *P. univalens* L3 atop equine intestinal epithelial cells.** Equine enteroid monolayers were grown in custom imaging chambers and monitored by live-cell microscopy during exposure to infective stage *P. univalens*, *S. vulgaris* and cyathostomin larvae. The movie shows a *P. univalens* L3 above the monolayer surface after two days co-incubation. Scale bar = 50 µm.

**Additional file 3. Live-cell imaging of a *S. vulgaris* L3 atop equine intestinal epithelial cells.** Equine enteroid monolayers were grown in custom imaging chambers and monitored by live-cell microscopy during exposure to infective stage *P. univalens*, *S. vulgaris* and cyathostomin larvae. The movie shows a *S. vulgaris* L3 above the monolayer surface after two days co-incubation. Scale bar = 50 µm.

## Data Availability

The data that support the findings of this study are available on request from the corresponding author.
