# Supplementary material for: Equine enteroid-derived monolayers recapitulate key features of parasitic intestinal nematode infection
Source: Vet Res. 2024 Feb 27;55:25. doi: 10.1186/s13567-024-01266-1 (PMC10900620; doi:10.1186/s13567-024-01266-1)
Supplement: Supplementary file 1 — Additional file 1. Table of novel primers for qPCR. [file 13567_2024_1266_MOESM1_ESM.docx]

| **Transcript** | **Primer sequence** | **Product size (bp)** | **Annealing temp (°C)** | **Primer konc (nM)** | **Efficiency %** |
| --- | --- | --- | --- | --- | --- |
| CXCL10 | F:GCACGCTGTACCTGCATTAA  R:GGATTCAGACATCTTTTCTCCCC | 151 | 59 | 400 | 95.2% |
| MIF | F:CAGCTCATGACTTTCGGCG  R:CCGCGTTCATGTCGTAGTAG | 180 | 59 | 400 | 97.5% |

**Additional file 1. Novel primers for qPCR**
